# Historical eye on IPF: a cohort study redefining the mortality scenario

**DOI:** 10.3389/fmed.2023.1151922

**Published:** 2023-06-02

**Authors:** Sara Tomassetti, Claudia Ravaglia, Sara Piciucchi, Jay Ryu, Athol Wells, Luca Donati, Alessandra Dubini, Catherine Klersy, Valentina Luzzi, Leonardo Gori, Elisabetta Rosi, Federico Lavorini, Venerino Poletti

**Affiliations:** ^1^Department of Clinical and Experimental Medicine, Interventional Pulmonology Unit, Careggi University Hospital, Florence, Italy; ^2^Pulmonary Unit, Department of Diseases of the Thorax, GB Morgagni Hospital, Forlì, Italy; ^3^Radiology Department, Ospedale GB Morgagni, Forlì, Italy; ^4^Respiratory and Critical Care Medicine, Mayo Clinic, Rochester, MN, United States; ^5^ILD Unit, Pulmonary Medicine, Royal Brompton Hospital, London, United Kingdom; ^6^Pathology Unit, Ospedale GB Morgagni, Forlì, Italy; ^7^Servizio di Biometria ed Epidemiologia Clinica, Fondazione IRCCS Policlinico San Matteo, Pavia, Italy; ^8^Department of Respiratory Diseases and Allergology, Aarhus University Hospital, Aarhus, Denmark

**Keywords:** idiopathic pulmonary fibrosis, interstitial lung disease, pirfenidone, nintedanib, cryobiopsy

## Abstract

**Rationale:**

Therapies that slow idiopathic pulmonary fibrosis (IPF) progression are now available and recent studies suggest that the use of antifibrotic therapy may reduce IPF mortality.

**Objectives:**

The aim of the study was to evaluate whether, to what extent, and for which factors the survival of IPF in a real-life setting has changed in the last 15 years.

**Methods:**

Historical eye is an observational study of a large cohort of consecutive IPF patients diagnosed and treated in a referral center for ILDs with prospective intention. We recruited all consecutive IPF patients seen at GB Morgagni Hospital, Forlì, Italy between January 2002 and December 2016 (15 years). We used survival analysis methods to describe and model the time to death or lung transplant and Cox regression to model prevalent and incident patient characteristics (time-dependent Cox models were fitted).

**Measurements and main results:**

The study comprised 634 patients. The year 2012 identifies the time point of mortality shift (HR 0.58, CI 0.46–0.63, *p* < 0.001). In the more recent cohort, more patients had better preserved lung function, underwent cryobiopsy instead of surgery, and were treated with antifibrotics. Highly significant negative prognostic factors were lung cancer (HR 4.46, 95% CI 3.3–6, *p* < 0.001), hospitalizations (HR 8.37, 95% CI 6.5–10.7, *p* < 0.001), and acute exacerbations (HR 8.37, 95% CI 6.52–10.7, *p* < 0.001). The average antifibrotic treatment effect estimated using propensity score matching showed a significant effect in the reduction of all-cause mortality (ATE coeff −0.23, SE 0.04, *p* < 0.001), acute exacerbations (ATE coeff −0.15, SE 0.04, *p* < 0.001), and hospitalizations (ATE coeff −0.15, SE 0.04, *p* < 0.001) but no effect on lung cancer risk (ATE coeff −0.03, SE 0.03, *p* = 0.4).

**Conclusion:**

Antifibrotic drugs significantly impact hospitalizations, acute exacerbations, and IPF survival. After the introduction of cryobiopsy and antifibrotic drugs, the prognosis of IPF patients has significantly improved together with our ability to detect IPF at an earlier stage.

## Introduction

Idiopathic pulmonary fibrosis (IPF) is a chronic fibrosing interstitial lung disease of unknown etiology associated with the radiological and/or histological pattern of usual interstitial pneumonia, with a median survival time of 3–5 years from diagnosis (1). Lung function declines in all IPF patients, and acute exacerbations or lung cancer can occur at any time in the disease course, increasing healthcare resource utilization, hospitalizations, and ultimately leading to death (2–4).

During the last decade, clinical trials have led to the approval of pirfenidone and nintedanib, the first two antifibrotic drugs with a specific indication for IPF (5, 6). Clinical trials have demonstrated that nintedanib and pirfenidone reduce lung function decline in IPF patients, with significant effect across subjects with both preserved forced vital capacity (FVC) (7, 8) and more advanced disease (9). IPF has a mortality rate that is similar to that of several cancers, and all-cause mortality would be the most clinically meaningful and preferred primary outcome in treatment trials. However, the necessary size, duration, and cost of mortality-powered studies in mild-to-moderate IPF are substantial and prohibitive (10); therefore, efficient clinical trials of feasible size and duration have been designed to show mainly to slow disease progression. Recently, a growing body of evidence has shown that pirfenidone and nintedanib reduce the risk of acute deterioration in lung function and improve life expectancy (11–13). Pooled analysis, meta-analysis, and IPF registries have shown that pirfenidone reduces the risk of death (14–16). However, the strength of those recent studies is hampered by methodological limitations. All-cause mortality in the trial population was low (3.1% in the pirfenidone and 6.5% in the placebo pooled group) and to what extent the results of this highly selected population followed using a rigid research methodology are generalizable to real-world patients remained unclear (17). A recent study evaluated survival trends in the United States showing a decline in IPF-related mortality from 2004 to 2017, but factors related to this mortality change remained unexplained (18). Real-world experiences of IPF registries report a mortality reduction similar to what was observed in pooled analysis and meta-analysis, but fail to capture the temporal trend in overall survival, do not include all possible covariates that might be associated with survival including time-dependent intermediate factors as hospitalizations, acute exacerbations, and lung cancer and do not apply a matching strategy to adjust for the inevitable heterogeneity of a real-life IPF populations (11, 19, 20).

The concern for adverse events and a quote of skepticism on antifibrotic efficacy are still rooted in some pulmonary physicians, leading to the possible risk of delayed treatment (21).

In this study, we report 15 years of real-life clinical experience, which to our knowledge is the largest monocentric prospective IPF cohort ever published. This study was designed to identify the shift in IPF patients' mortality observed in recent years and the factors that have driven this change. The primary objective was to define overall survival and to identify any relevant change in survival trends. The secondary objectives were to identify the clinical and demographic prognostic factors contributing to the observed shift in mortality.

## Methods

### Study design and patient selection

This is an observational cohort single-center study that follows the STROBE guidelines. We recruited all consecutive patients who received a multidisciplinary diagnosis of IPF at GB Morgagni Hospital, Pulmonary Unit, between January 2002 and December 2016 (15 years). The quality of data was improved by the introduction of the database of standardized data forms and internal checks, and follow-up data were collected with prospective intention. The expert multidisciplinary team used both ATS/ERS guidelines and Fleischner Society white paper criteria (1, 22, 23). All data were extracted from the Pulmonary Unit ILD database in which patients were enrolled using a standardized initial assessment (function, HRCT, BAL, laboratory tests including autoimmunity in all cases, and biopsy in selected cases) and a follow-up structured with prospective intention (visit every 4–6 months with clinical and functional evaluations, HRCT, and echocardiography on a yearly basis). We extracted clinical information from medical records using a standard form, as detailed in the Supplementary material, page 2.

The study was approved by the Comitato Etico di area vasta ROMagna, Italy (CEROM approval protocol number 8571/2017). Patients provided informed consent according to current local and national legislation.

### Outcomes

The primary outcome was to define overall survival, identifying any relevant change in survival trends and the time point at which the mortality switch occurred. We compared survival stratified by year of diagnosis, and we characterized two distinct historical cohorts with different survival profiles. Clinical and demographic factors considered clinically relevant and/or potentially associated with survival were analyzed in order to identify the factors contributing to the observed shift in mortality.

### Statistical analysis

Continuous data were described with the mean and standard deviation (SD) and categorical data as counts and percent. We used Student's *t*-test and the chi-square test to compare groups of interest. We used survival analysis methods to describe and model the time to death or lung transplant, including rates per 100 person years and 95% confidence intervals (95% CI) and Kaplan–Meier cumulative event-free survival and 95% CI to describe; we used Cox regression to model prevalent and incident patients characteristics; in this case, time-dependent Cox models were fitted. We computed hazard ratios (HR) and 95% CI. We assessed graphically the proportional hazard assumptions by plotting the observed and predicted survival curves. Multivariable models included non-collinear variables that were considered of clinical relevance and gave a signal at the univariate analysis (*p* < 0.2). The Harrell's C statistic was computed to assess discrimination. We also computed the average treatment effect after propensity score matching.

No imputation of missing data was performed given the low number of missing data in the variables of interest.

Propensity-score-based matching was used to select control patients similar to patients receiving treatment (matched by age, gender, comorbidities, and pulmonary function—% of pred. FVC and % of pred. DLco).

All statistical analyses were performed using STATA 15 (StataCorp, College Station, TX, USA).

### Role of funding source

This investigator-initiated study was funded by F. Hoffmann-La Roche Ltd; Genentech, Inc. The sponsor had no role in the study design, data collection, analysis, final report, and decision to submit for publication. ST and VP had full access to all the data in the study and had final responsibility for the decision to submit for publication.

## Results

### Population

In total, 634 patients were diagnosed within the prespecified protocol window (between the year 2002 and the year 2016) and met protocol requirements as detailed in the Supplementary material (Supplementary Figure S1, page 2 of the Supplementary material).

**Figure 1:**
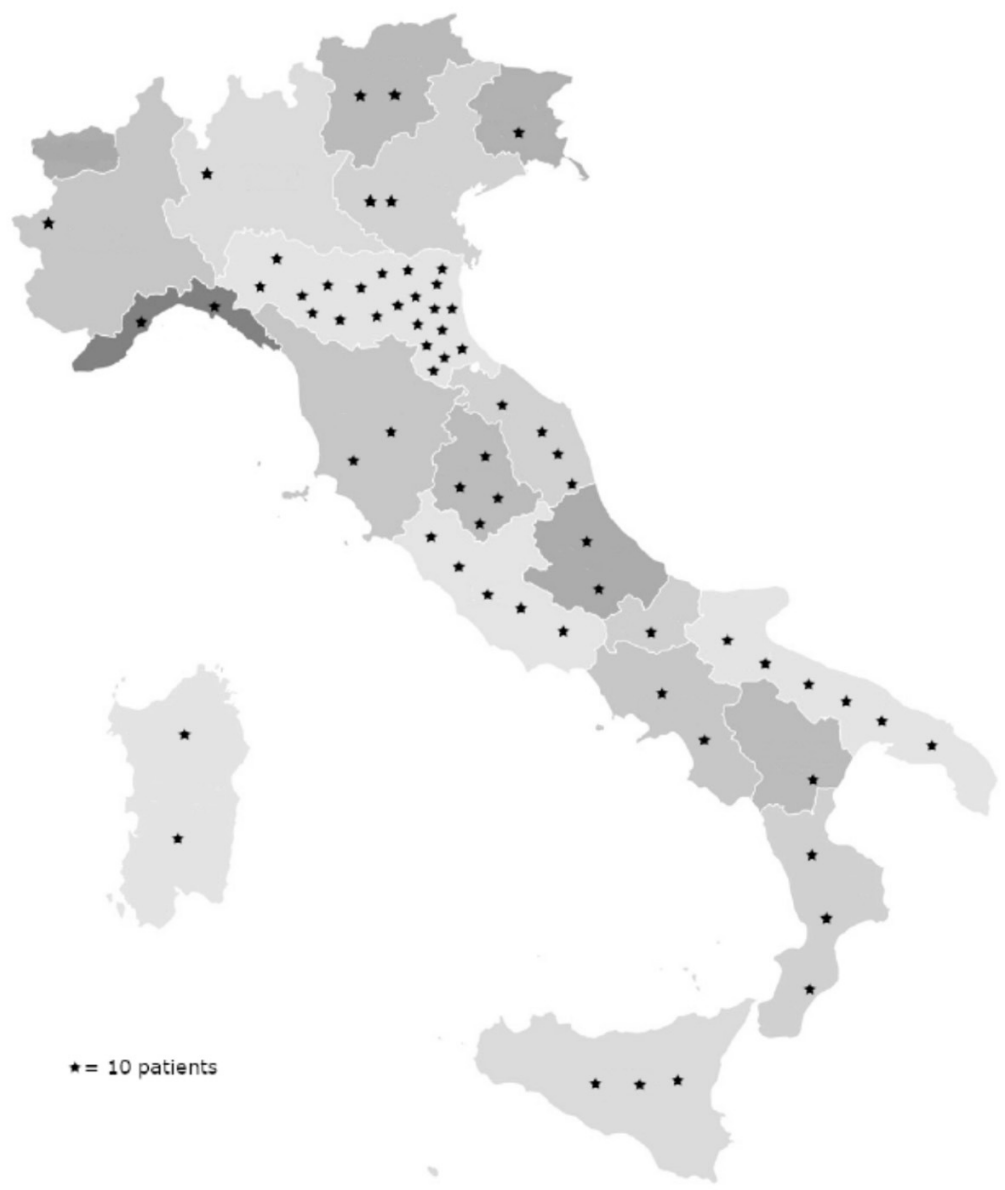
Geographical distribution of patients with the diagnosis of IPF made at our center. Numbers were approximated to 10 patients per star represented in the graph.

Approximately 99.7% (*N* = 632) of patients were Caucasian, and 0.3% (*N* = 2) were Hispanic (from South America). At the time of diagnosis, all patients were Italian residents, coming from all but one Italian region, mainly central and southern Italy (Figure 1). Patients' characteristics are summarized in Table 1.

**Table 1:** Clinical Characteristics of the patients according to diagnosis period.

	**Diagnostic period**				
	**Missing values**	**Entire cohort**	**(2002–2011)**	**(2012–2016)**	***p*** **-value**
		***N*** = **634**	***N*** = **286**	***N*** = **348**	
Age (yr.)		66.8 ± 8.6	65.6 ± 8.8	67.7 ± 8.3	0.0003
Gender N (%) of male patients		478 (75.4)	219 (76.6)	259 (74.4)	0.53
Family history of ILDs		127(20)	55 (19.2)	72 (20.7)	0.65
Current Former Smokers N (%)	10	444(71.1)	211 (74.8)	233 (68.1)	0.04
Pack years	288	30.6 ± 22.2	31.3 ± 21.5	30.0 ± 22.9	0.29
Comorbidities. N (%)		536(84)	236 (82.5)	300 (86.2)	0.20
Emphysema		115(18.1)	53 (18 5)	62 (17.8)	045
Lung cancer		61(9)	36 < 12 (6)	25 (7.2)	0.016
Pulmonary hypertension	204	219(50)	133 (62.7)	86 (39.4)	< 0.0001
Body Mass Index	20	27.7 ± 4.1	27.8 ± 4	27.5 ± 4.2	0.45
Functional measures					
FVC (% of predicted)	5	77.3 ± 19.2	74.5 ± 19.8	79.6 ± 18.4	0.0005
FEV1 (% of predicted)	8	84.4 ± 20. 4	80.3 ± 20.6	87 ± 19.7	< 0.0000
DLco (% of predicted)	13	50.1 ± 16.6	47.4 ± 16.7	52 4 ± 16.2	0.0001
Use of oxygen under exercise N (%)	26	143(23.5)	76 (28.6)	67 (19.6)	0.006
mMRC	171	1.7 ± 0.8	1.8 ± 0.7	1.6 ± 0.8	0.003
6min walking test distance (m)	270			385.5 ± 123.8	0.08
Diagnostic procedures			SD ±		
HRCT definite UIP N (%)		406 (64)	195(68)	21(60)	0.029
Lung cryobiopsy N (%)		141 (22)	20 (7.0)	121 (34.8)	< 0 000
Surgical lung biopsy N (%)		95 (15)	73 (25.5)	22 (6.3)	< 0.000
Patients with BAL lymphocytosis >30% N (%)	184	24 (5.3)	13 (6.7)	11 (43)	0.18

Plus-minus values are means ± SE. Abbreviations: FEV1, forced expiratory volume in one second; FVC, forced vital capacity; DLco, carbon monoxide diffusion capacity; ILDs, interstitial lung diseases. P values are calculated with Mann-Whitney test) and compare the two time periods (2002–2011 to 2012–2016). Comorbidities denotes the number of patients with one or more comorbidity excluding emphysema, lung cancer and pulmonary hypertension. 6 min WT was conducted with or without oxygen supplementation as needed. Pulmonary hypertension likelihood was extimated by echocardiography.

### Overall survival trends over time

Of the 634 IPF patients included in the study, definite outcome data (date of death or last known visit) were available for all cases. At the time of censoring the data, 335 patients had died (52.8%) and 25 had been transplanted (3.9%). Overall median survival was 4.67 years (25th−75th percentile 2.26–8.07 years). For the 360 patients who died or were transplanted, the median time to event was 2.71 years (25th−75th percentile 1.5–14.48 years). The rates for 1-year, 3-year, 5-year, and 8-year survival were 90% (SE 0.011, CI 0.74–0.81), 67% (SE 0.19, CI 0.643–0.70), 46% (SE 0.02, CI 0.42–0.50), and 26% (SE 0.026, CI 0.21–0.31), respectively. The estimated rate of death was 15.12 (95% CI 13.63–16.75) per 100 persons per year. Supplementary Figure S2 page 3 of the Supplementary material shows the overall survival of the IPF population.

**Figure 2:**
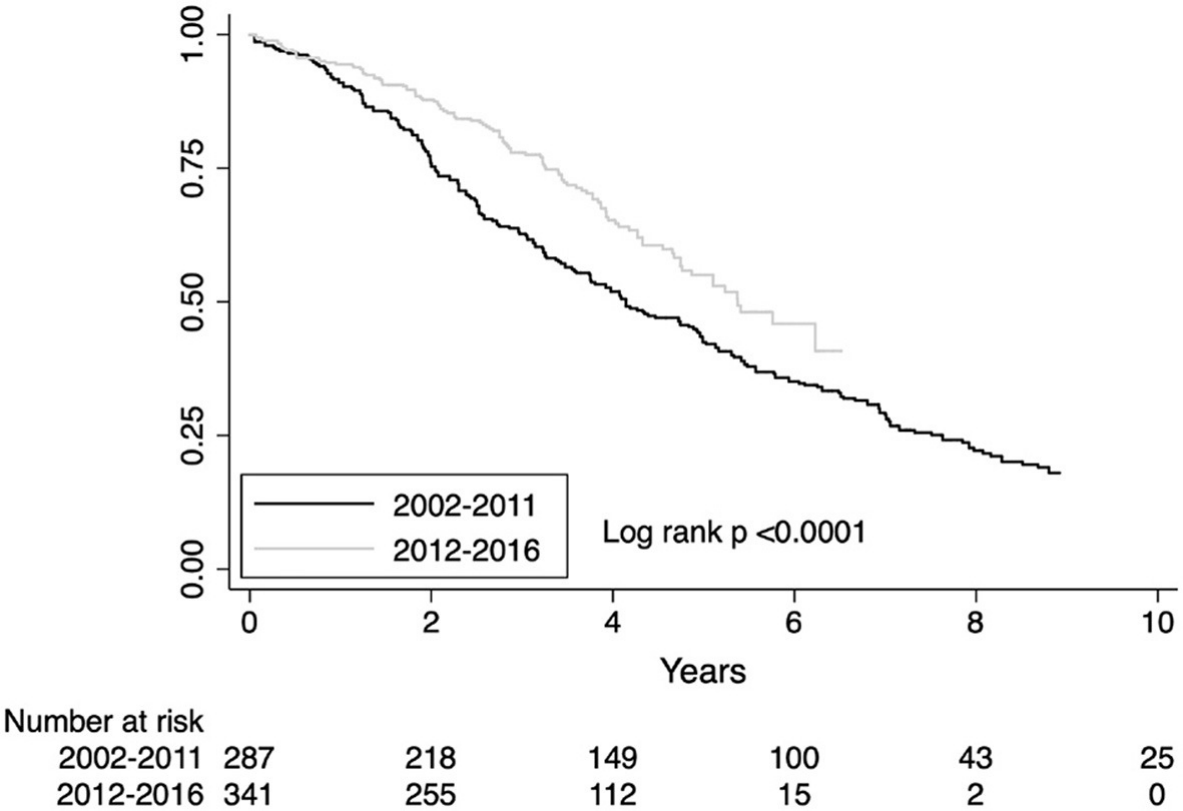
Survival estimates for the diagnostic period before or after 2012.

To model survival trends over the period of diagnosis, we split the population both by calendar year of diagnosis and by grouping patients every 3 years (2002–2004; 2005–2007; 2008–2010; 2011–2013; 2014–2016) and 5 years (2002–2206; 2007–2011; 2012–2016). The difference in mortality risk was not statistically significant when stratified by calendar year (survival trends are shown in Supplementary Figure S3 page 4 of the Supplementary material). When stratified every 3 years, the mortality declined in the last triennium (2014–2016), unadjusted HR 0.60, CI 0.37–0.98; *p* = 0.04 but was not significant after adjusting for age, gender, FVC, DLco HR 0.66, CI 0.40–1.10; *p* = 0.40 (Supplementary Figure S4 page 5 of the Supplementary material). For the 3-year time point, the estimated area under the ROC curve was 0.52. The sharpest reduction in mortality was observed stratifying patients every 5 years. The observed reduction in mortality risk in the last quinquennium (2012–2016) was HR 0.58, CI 0.43–0.79 (unadjusted), *p* < 0.001 and HR 0.63, CI 0.46–0.86, *p* < 0.001 after adjusting for covariates (Supplementary Figure S5 page 6 of the Supplementary material). For the 5-year time point, the estimated area under the ROC curve was 0.39. In our patients' cohort, the year 2012 identifies the time point of fracture from the past. Survival difference for the diagnostic period before and after the year 2012 is shown in Figure 2. The observed reduction in mortality risk for the diagnostic period before and after 2012 was HR 0.58, CI 0.46–0.63, *p* < 0.001 (unadjusted) and HR 0.66, CI 0.52–0.83, *p*= 0.001 after adjusting by age, gender, FVC, and DLco.

### Over-time changes in IPF clinical profile

To identify clinical factors that may have changed over time, we compared clinical characteristics of IPF cases diagnosed in the last 5-year period (2012–2016) to those diagnosed in the first decade (2002–2011), as reported in Table 1. Compared to the first decade, patients who underwent an IPF diagnosis after 2012 were slightly older. Overall, the burden of comorbidities did not change over time, including emphysema, but the prevalence of both lung cancer (from 12.6% to 7.2%, *p* = 0.016) along with the likelihood of pulmonary hypertension (62.7% to 39.4%, *p* < 0.0001) significantly decreased. Patients diagnosed in the last 5 years had a slightly better preserved lung function, as measured by % of predicted FVC (74.5% and 79.6%, respectively), % predicted FEV1 (80 and 87.0, respectively), and % predicted DLco (47.4 and 52.4, respectively) (*p* < 0.001 in all three groups comparison). Accordingly, the dyspnea index (1.8 and 1.6, *p* < 0.003) and the use of oxygen under exercise (28.6% and 19.6%, *p* < 0.006) were significantly less frequent in patients diagnosed in the last quinquennium compared to the previous decade. In the last 5 years at our center, we have observed a significant increase in the total number of IPF diagnoses (from 286 to 348), along with an increased prevalence of cases confirmed by lung biopsy (from 31.8% to 39.4%, *p* < 0.049). This escalation has been mainly driven by the implementation of transbronchial lung cryobiopsy (TBLC, up to 34.8% of cases) which has minimized the role of surgical lung biopsy (SLB, down to 6.3% of cases).

### Potential prognostic factors

#### Univariate analysis of potential clinical prognostic factors

Univariate analysis suggested that the time period of diagnosis (before/after 2012), age, pulmonary hypertension and lung cancer, functional impairment (% pred FVC, % pred FEV1, % pred DLco), grade of dyspnea (mMRC), use of oxygen under exercise, and diagnosis confirmed by cryobiopsy were all significant prognostic factors for overall survival (*p* < 0.05), as shown in Table 2.

**Table 2:** Statistical distributions of potential prognostic factors.

	**Univariate analysis**			**Multivariate analysis**	
	**Rate per 100p/y**	**HR (95% CI)**	***p*** **value**	**HR (95% CI)**	***p*** **-value**
Period of diagnosis (2012–2016)	10.6 (8.8–12.7)	0.58 (0 46–0.73)	< 0.0000	0.58 (0.40–0.83)	0.003
Age (years)	16.4 (14.4–18.8)	1.02 (0.01–1.03)	0.0008	1.02 (1–1.04)	0.18
Gender Male	15.6 (13.8–17.3)	1.14 (0.89–1.46)	0.28	1.09 (0.7–1.6)	0.65
Family history of ILDs	13.14 (10.6–16.9)	0.83 (0.6–1.1)	0.15	–	–
Current former smokers	15.8 (614–17.8)	1.19 (0.94–1.50)	0.15	–	–
Pack years		1.01 (1–1.02)	0.42	–	–
Comorbidities^∧^	14.8 (13.2–16.5)	1.08 (0.99–1.18)	0.09	–	–
Emphysema	15.7 (12.3–20)	1.07 (0.8–1.4)	0.62	–	–
Lung cancer	24.8 (18.8–32.9)	1.78 (1.32–2.41)	**0.0001**	2.24 (1.38–3.63)	**0.001**
Pulmonary hypertension	17.5 (14.9–20.5)	1.57 (1.21–2.04)	**0.0006**	0.98 (0.69–1.39)	0.9
Body mass index	–	0.99 (0.97–1.02)	0.68	–	–
**Functional measures**					
FVC (% of predicted)	–	0.97 (0.97–0.98)	**< 0.0000**	0.95 (0.92–0.98)	**0.002**
FEV1 (% of predicted)	–	0.98 (0.97–0.98)	**< 0.0000**	1.03 (1–1.06)	**0.041**
DLco (% of predicted)	–	0.95 (0.95–0.96)	**< 0.0000**	0.96 (0.94–0.97)	**< 0.000**
Use of oxygen under exercise	27.6 (23–33–1)	2.57 (82.05–3.2)	**< 0.0000**	1.2 (0.9–1.7)	0.85
mMRC & 2	19.1 (16.5–22.1)	1.7 (1.3–2.2)	**< 0.0000**	0.89 (0.6–1.25)	0.5
**Diagnostic procedures**					
Lung cryobiopsy	5.8 (4.05–8.4)	0.34 (0.23–0.5)	**< 0.0000**	1.06 (0.6–1.8)	0.8
Surgical lung biopsy	15.2 (12.1–19.3)	0.94 (0.72–1.23)	0.65	–	–
Patients with BAL lymphocytosis >30%	15.6 (9.4–25.8)	1.13 (0.67–1.91)	0.64	–	–

*Rate per 100 persons year if Age > 65. ^∧^Comorbidities number excluding emphysema, lung cancer and pulmonary hypertension; rate per 100-person year was calculated for patients with one or more comorbidity. Bold values are highlighted in bold to distinguish them as statistically significant values from those that are not statistically significant.

#### Multivariable analysis of potential clinical prognostic factors

Table 2 shows the results of the Cox regression analysis. There was a significantly greater risk of mortality associated with the diagnosis period (before/after 2012), the presence of lung cancer, and functional impairment as measured by % of predicted FVC, % of predicted FEV1, and % of predicted DLco. The presence of family history for ILDs, age, gender, smoking history, comorbidities (excluding lung cancer), use of oxygen under exercise, grade of dyspnea, and diagnosis confirmed by cryobiopsy were non-significant when adjusted for covariates.

#### Intermediate potential prognostic factors: lung cancer, hospitalizations, acute exacerbations, and disease progressions

Lung cancer, hospitalizations for any cause, and acute exacerbations were all less frequent in the last quinquennium compared to the previous decade (lung cancer *N* = 25, 7% compared to 36, 13%, *p* = 0.016; hospitalizations *N* = 115, 52% compared to *N* = 56, 19%, *p* < 0.001; acute exacerbations *N* = 36, 12.5% compared to *N* = 87, 40%, *p* < 0.001). The prevalence of patients who experienced a disease progression was higher in cases diagnosed after the year 2012 (*N* = 225 43.5% before 2012 compared to *N* = 292, 56.5% after 2012), but more patients had only one progression in this group (1POD *N* = 251, 85%; 2 POD *N* = 22, 7.5% 3POD *N* = 4, 1.4% diagnosis after 2012), whereas, in the cohort of patients diagnosed before 2012, the number of patients with multiple POD was significantly higher (1POD *N* = 140, 62%; 2 POD *N* = 25, 20%; 3 or more POD *N* = 18, 8%).

Time-dependent Cox regression analysis showed that intermediate factors (i.e., lung cancer, hospitalizations, acute exacerbations, and disease progressions) were all significantly associated with a greater risk of mortality. Lung cancer increased mortality risk HR 4.46 (95% CI 3.3–6), *p* < 0.001. Patients with one or more all-cause hospitalizations showed an increased risk of death HR 7.20 (95% CI 5.64–9.20), *p* < 0.001. Similarly, patients with one or more acute exacerbations had a significantly increased risk of death HR 8.37 (95% CI 6.52–10.7), *p* < 0.001, and patients with one or more disease progression at follow-up had a significantly increased risk of death HR 9.08 (95% CI 5.8–14.2), *p* < 0.001.

### Treatment

Idiopathic pulmonary fibrosis treatment at our center significantly changed after 2012. As shown in Table 3, the use of antifibrotic has completely overcome both the previously common immunosuppressive treatment with azathioprine and the less common use of cyclophosphamide. The use of both corticosteroids and Warfarin has been reduced to one-third in this fragile population.

**Table 3:** Treatment approaches according to diagnosis period.

	**Entire cohort**	**(2002–2011)**	**(2012–2016)**	***p*-value**
	***N*** = **634**	***N*** = **286**	***N*** = **348**	
Pirfenidone, *N* (%)	273 (43)	76 (27)	197 (57)	< 0.001
Nintedanib, *N* (%)	112 (18)	18 (6)	94 (27)	< 0.001
Immunosuppression, *N* (%)	99 (16)	88 (30)	11 (3)	< 0.001
Azathioprine, *N*	90	80	10	
Cyclophosphamide, *N*	9	8	1	
Corticosteroids only, *N* (%)	142 (22)	98 (34)	44 (12)	< 0.001
Warfarin, *N* (%)	36 (6)	26 (9)	10 (3)	0.001

In the subgroup of patients treated with antifibrotics compared to any other treatment, the number of hospitalizations for any cause was significantly reduced (45% vs. 65%, *p* < 0.001) and the reduction was significant with both antifibrotic drugs: pirfenidone 36% vs. 56%, *p* < 0.001 and nintedanib 16% vs. 23%, *p* < 0.052. Similarly, acute exacerbations were less frequent in patients treated with antifibrotic drugs (39% vs. 64%, *p* < 0.001) and in both treatment subgroups (pirfenidone 32% vs. 54%, *p* < 0.001 and nintedanib 11% vs. 24 %, *p* < 0.003). Whereas, both hospitalizations and acute exacerbations were more frequent in patients treated with immunosuppressive drugs compared to any other treatment regimen (hospitalizations 22% vs. 12%, *p* < 0.002 and acute exacerbations 24% vs. 13%, *p* < 0.002).

**Figure 3:**
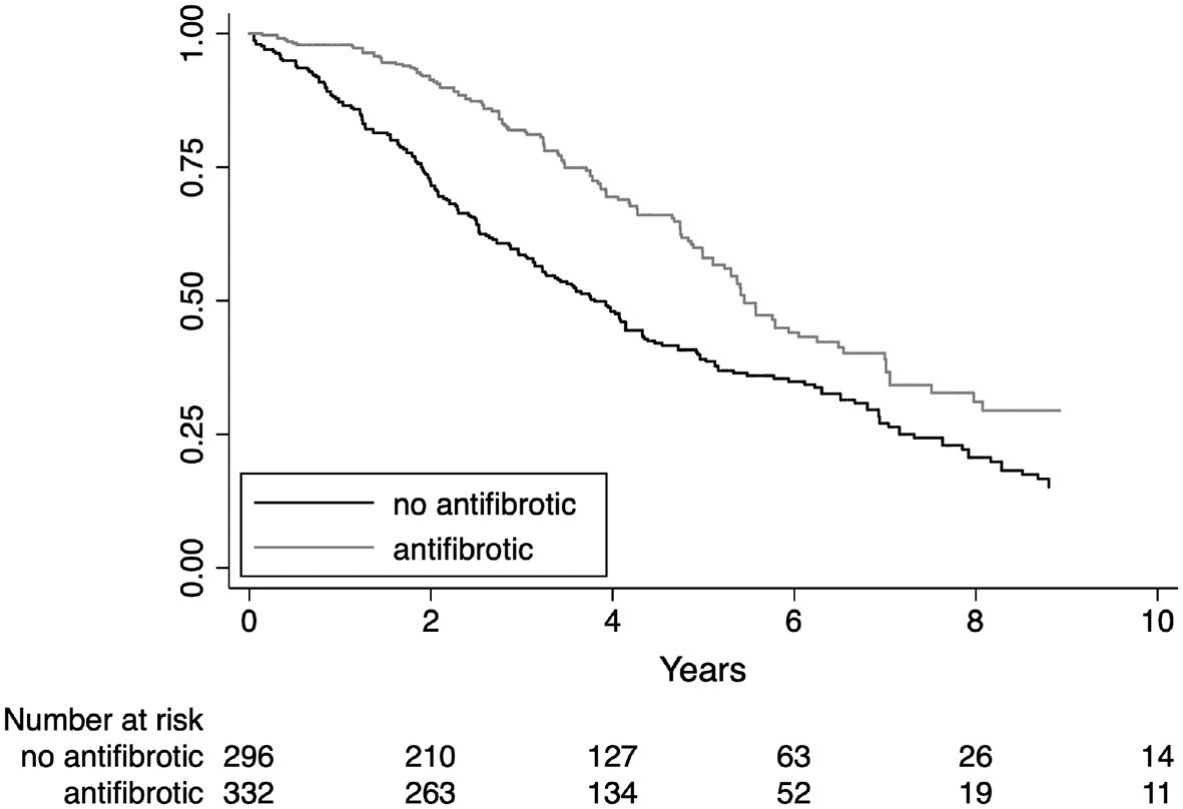
Survival estimates by treatment.

Kaplan–Meier survival estimates for treatment subgroups are shown in Figure 3. The average antifibrotic treatment effect coefficient estimated by propensity score matching showed a significant reduction in mortality rate, acute exacerbations, and hospitalizations as shown in Table 4. Immunosuppressive treatment showed a significant increase in mortality, acute exacerbations, and hospitalizations. The propensity score matched analysis did not identify any significant correlation between the use of antifibrotics or immunosuppressive treatment and the incidence of lung cancer (data shown in the Supplementary Table S1, page 7).

**Table 4:** Impact of treatment on mortality, acute exacerbations, and hospitalizations.

**Outcome**	**Treatment**	**ATE coeff**	**SE**	***p*-value**
Death and lung transplant	Any antifibrotic	−0.23	0.04	**< 0.001**
	Pirfenidone	−0.24	0.04	**< 0.001**
	Nintedanib	−0.2	0.001	**0.001**
	Any immunosoppressive agent	0.36	0.05	**< 0.001**
	Corticosteroids only	0.2	0.07	0.06
Acute Exacerbations	Any antifibrotic	−0.15	0.04	**< 0.001**
	Pirfenidone	−0.13	0.05	**0.006**
	Nintedanib	−0.06	0.05	0.2
	Any immunosoppressive agent	0.13	0.06	**0.040**
	Corticosteroids only	0.1	0.08	0.2
Hospitalizations	Any antifibrotic	−0.15	0.05	**0.002**
	Pirfenidone	−0.19	0.05	**< 0.001**
	Nintedanib	−0.01	0.05	0.800
	Any immunosoppressive agent	0.12	0.06	**0.049**
	Corticosteroids only	0.22	0.08	**0.006**

ATE, average treatment effect; SE, AI robust standard error; CI, confidence interval.

The average treatment effect was estimated using propensity score matching (covariates: age, % of predicted FVC, % of predicted DLco, and lung cancer). Bold values are highlighted in bold to distinguish them as statistically significant values from those that are not statistically significant.

## Discussion

The year 2012 identifies the time point of fracture from the past, with a significant reduction in mortality risk for IPF (HR 0.58, 95% CI 0.46–0.63). Among all the clinical factors that have been evaluated in this study, a more preserved lung function and antifibrotic treatment are the only determinants of a better prognosis, whereas lung cancer and immunosuppressive treatment are related to a worse survival. The year 2012 at our center coincided with both the introduction of antifibrotic treatment and transbronchial lung cryobiopsy (TBLC) for the diagnosis of ILDs that significantly increased patients' referrals to our unit and the volume of new IPF cases identified.

Some clinical differences noted between the two different IPF time groups are of speculative interest. In the most recent cohort (i.e., after 2012), baseline lung function is more preserved, and this can have contributed to the observed lower prevalence of pulmonary hypertension and lung cancer, comorbidities that tend to occur at more advanced stages. The timely management of patients is a real hope for the future, given the negative prognostic impact that a delayed diagnosis and treatment have (24). Neither familial IPF nor lymphocytosis on BAL had a meaningful impact on mortality risk. Based on our preliminary findings, we can postulate that those variables should not influence physicians' diagnostic perception, nor delay the treatment decision. However, we recognize the limits of this retrospective single-center study, and we believe that this issue should be more appropriately investigated in a prospective analysis.

Antifibrotic treatment has clearly shown in phase III randomized and controlled clinical trials to slow disease progression (5, 6). The pooled analyses of CAPACITY, ASCEND, and INPULSIS trials have shown that nintedanib can reduce the incidence of acute exacerbations (5) and pirfenidone can reduce both respiratory hospitalizations (25) and mortality (14). The population of those clinical trials may significantly diverge from what we observe in real life where patients are older, sicker, and often with numerous comorbidities (14, 17). The first large real-world observational retrospective PS-matched study that compared overall survival in treated and non-treated patients showed reduced all-cause mortality in the treated cohort, but left many open questions about the impact of antifibrotics on clinically relevant intermediate events such as acute exacerbations and lung cancer (26). Our study corroborates previous findings showing a significant mortality risk reduction for both antifibrotic treatments, pirfenidone and nintedanib, and explores the possible impact of antifibrotics on acute exacerbations and lung cancer. In the antifibrotic-treated patients, we observed a significant reduction in the risk of acute exacerbations, and hospitalizations for any cause. Combining all evidence, we can hypothesize that the mortality reduction observed in antifibrotic-treated patients is the result not only of the slowed functional decline, but also of the prevention of acute exacerbations leading to hospitalizations, for both drugs. The lack of statistical significance of the average treatment effect for nintedanib in the PS-matched analysis should be interpreted with great caution due to the significantly smaller number of patients in this treatment group compared to pirfenidone. Despite the lower lung cancer prevalence observed in recent years (7.2% vs. 12.6%, *p* = 0.016), the propensity score analysis showed no effect of antifibrotic treatment on lung cancer incidence, and the lower prevalence of lung cancer may merely reflect the milder disease observed in the recent cohort. Our findings do not support the results of previous retrospective studies, conducted on small series of patients (83 treated with pirfenidone, only two cases of lung cancer observed vs. 178 not treated, *N* = 39 lung cancer observed), that showed a dramatically decreased incidence of lung cancer in patients treated with pirfenidone (2.4% vs. 22%) and a decreased risk on multivariate analysis (HR 0.11, 95% CI 0.03–10.46) (27). We can hypothesize that the absence of a matched analysis may have hampered those preliminary results, but larger prospective multicenter studies should better address this important issue in the future.

The propensity score-matched analysis showed a clear increased mortality risk for IPF patients treated with immunosuppressive drugs, mostly azathioprine combined with steroids, whereas no effect on mortality was observed for patients treated with steroids only. When in 2012 the PANTHER trial revealed that patients in the combination therapy (prednisone–azathioprine–NAC), compared with the placebo group, had an increased rate of death (8 vs. 1, *p* = 0.01) and hospitalization (23 vs. 7, *p* < 0.001), the triple therapy approach was abandoned in clinical practice (28). However, some skepticism permeated the scientific community based on uncertainty about whether the reported adverse findings were specific to the use of azathioprine or the combination of azathioprine and steroid therapy, that in the trial was used at higher doses compared to clinical practice (29). Of note, although increased mortality was observed in patients receiving triple therapy, no functional worsening was found in this group, and roughly, one-third of patients receiving the combination therapy discontinued all three medications (30). Our results finally shed light on these areas of uncertainty confirming that the increased mortality risk observed for azathioprine treatment in IPF is linked to an increased risk of acute exacerbations and that the previously reported adverse findings apply at large in IPF. A recent study by Alqalyoobi et al. reported a decrease in in-hospital mortality for IPF patients admitted in academic hospitals (all-cause mortality, respiratory failure-associated mortality, and mechanical ventilation-associated mortality), but not for those admitted in non-academic hospitals. The major limits were the use of an administrative database (NIS) and the lack of data about antifibrotic treatment. The reasons of the observed difference remain unclear. However, the authors suggest a possible stronger adherence to 2015 IPF treatment guidelines at academic centers and our data corroborate the hypothesis that antifibrotic treatment could have influenced the observed decrease in in-hospital mortality for IPF patients admitted in academic hospitals (31).

All intermediate potential prognostic determinants (i.e., lung cancer, acute exacerbations, hospitalizations for any cause, and disease progression) showed a clear correlation with higher mortality risk at time-dependent analysis. Our results are in line with previous studies that documented an increased mortality risk for coexisting lung cancer (HR 5, 95% CI 2.91–8.57) (4), acute exacerbations (in-hospital mortality 50–90%) (3), respiratory hospitalizations (HR 6.22, 95% CI 4.07–9.49), and disease progression (HR 7.06, 95% CI 4.21–11.84) (32, 33).

Our study has several limitations. Although the cases were followed with prospective intention, real-world database management decisions for each patient were based on individual clinical practice rather than the trial protocol. Second, because this was not a clinical trial, we cannot make accurate comparisons between different treatment groups. Importantly, while most patients reported starting antifibrotic medications at baseline, some patients reported starting when the drug became available. Additionally, start times as well as medication use and comorbidities are self-reported and their accuracy may be limited. The number of treated patients between pirfenidone and nintedanib at that time was imbalanced in favor of pirfenidone because that was the drug that hit the market first in our region.

In conclusion, this large monocentric study investigates for the first time 15 years of real-life IPF history showing that the prognosis of our patients in the last 5 years has significantly improved and that both the introduction of antifibrotic treatment and the discontinuation of immunosuppressive drugs have significantly contributed to this change. The introduction of cryobiopsy and antifibrotic treatment at our site has coincided with a significant increase in patients' referrals and earlier diagnoses. The role of antifibrotic drugs in slowing functional decline, preventing acute exacerbations, and improving survival is confirmed in real-life settings. However, the preventive role of antifibrotic for lung cancer remains to be elucidated. Immunosuppression (azathioprine treatment combined with low doses of steroids) is proven to be harmful and should be discouraged in ascertained UIP/IPF patients. Low doses of steroids used alone do not seem to impact the prognosis of our patients and can be used in selected cases, carefully balancing advantages and possible side effects.

## Data availability statement

The original contributions presented in the study are included in the article/Supplementary material, further inquiries can be directed to the corresponding author.

## Ethics statement

The studies involving human participants were reviewed and approved by Comitato Etico di area vasta ROMagna, Italy (CEROM approval protocol number 8571/2017). The patients/participants provided their written informed consent to participate in this study.

## Author contributions

Conception and design: ST, CR, SP, and VP. Acquisition, analysis or interpretation and drafting the manuscript for important intellectual content: ST, SP, CR, AW, JR, AD, LD, CK, VL, LG, FL, ER, and VP. Full access to all of the data in the study and takes responsibility for the integrity of the data and the accuracy of the data analysis: ST and VP. All authors contributed to the article and approved the submitted version.
