# Supplementary material for: Historical eye on IPF: a cohort study redefining the mortality scenario
Source: Front Med (Lausanne). 2023 Jun 2;10:1151922. doi: 10.3389/fmed.2023.1151922 (PMC10273674; doi:10.3389/fmed.2023.1151922)

HE Supplement

Index

Figure S1 Enrolment flow chart ……………………………………………………………………………………………………..2

Figure S2 Overall survival of the IPF population………………………………………………………………………………..3

Figure S3 Event rate stratified by calendar year of diagnosis

Figure S4 Event rate stratified by calendar 3 years of diagnosis

Figure S5 Survival of the IPF population stratified by five years of diagnosis ………………………………….…6

Table S1 Propensity score matched analysis for treatment effect on lung cancer risk…………………….7

Figure S1 Enrolment flow chart

**IPF patients diagnosed between 1/1/2002 and 31/12/ 2016**

**N = 703**

**IPF patients final cohort**

**N = 634**

not IPF N= 8

lack of critical data N=  32

lost at follow up N= 29

*Critical data for enrolment were: multidisciplinary diagnosis of IPF, date of death or last known follow-up, HRCT, pulmonary function tests and clinical history at baseline available for revision.

Figure S2 Overall survival of the IPF population.


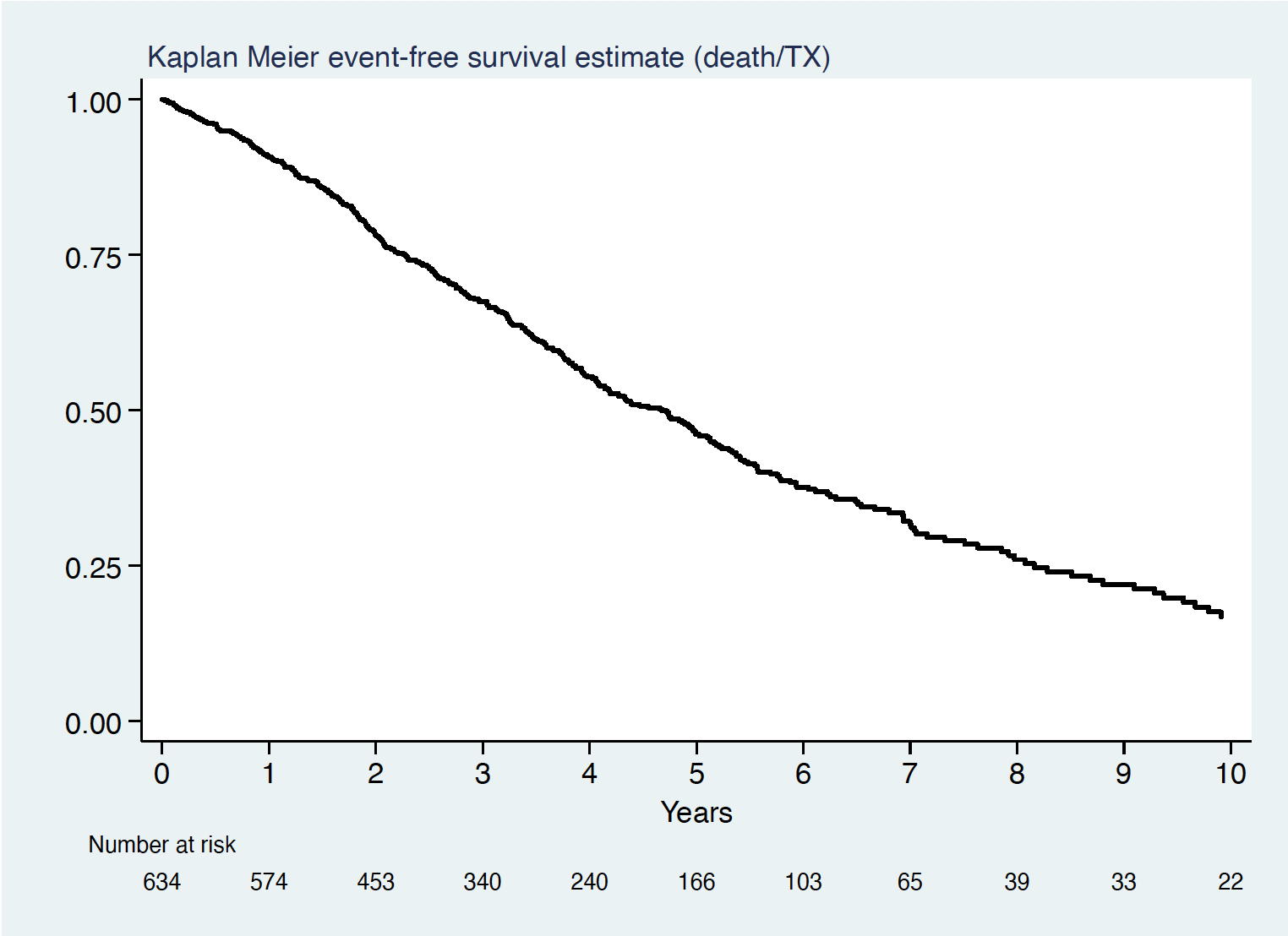


Figure S3 Event rate stratified by calendar year of diagnosis

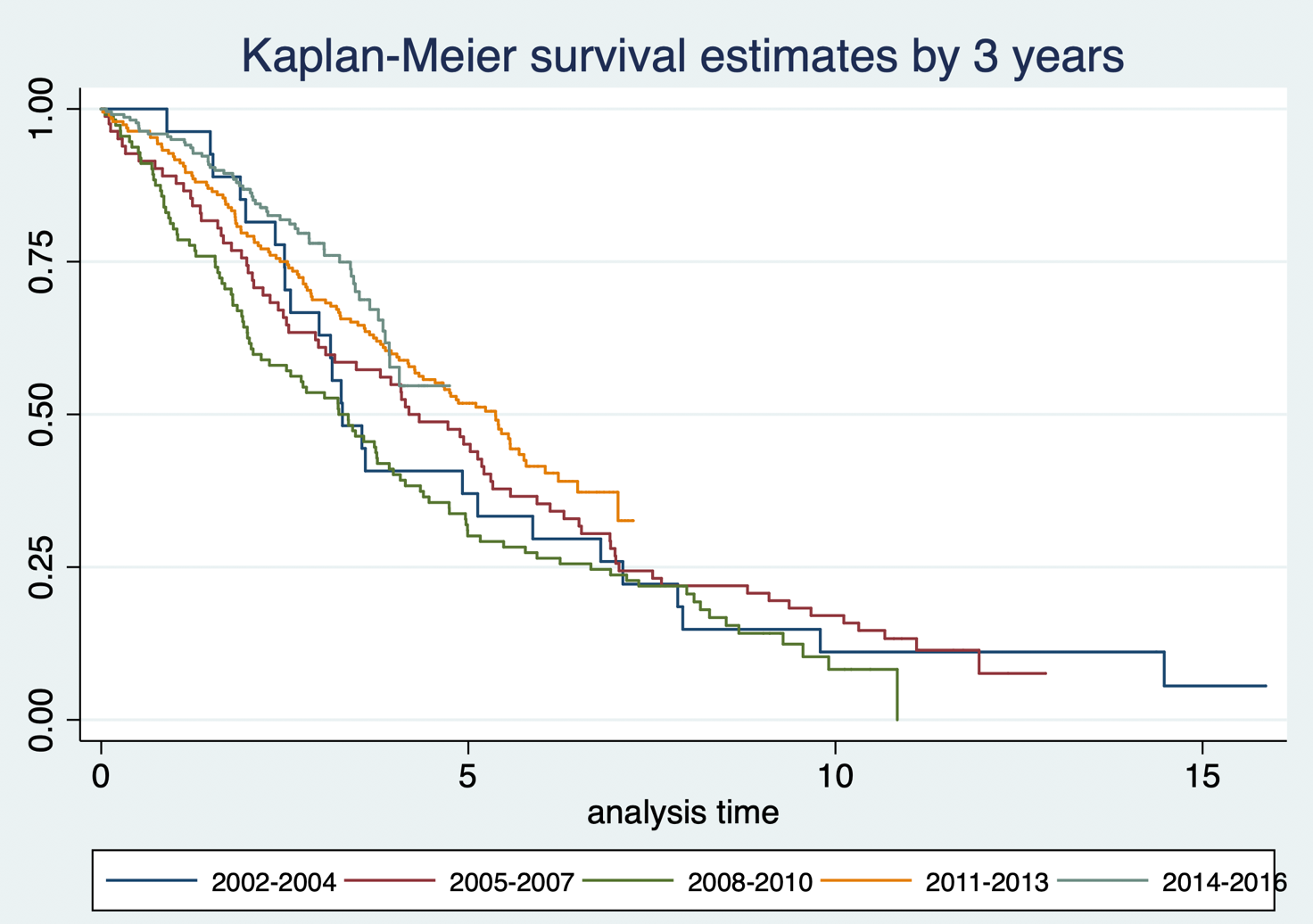


Figure S4 Event rate stratified by calendar 3 years of diagnosis

Figure S5 Survival of the IPF population stratified by five years of diagnosis


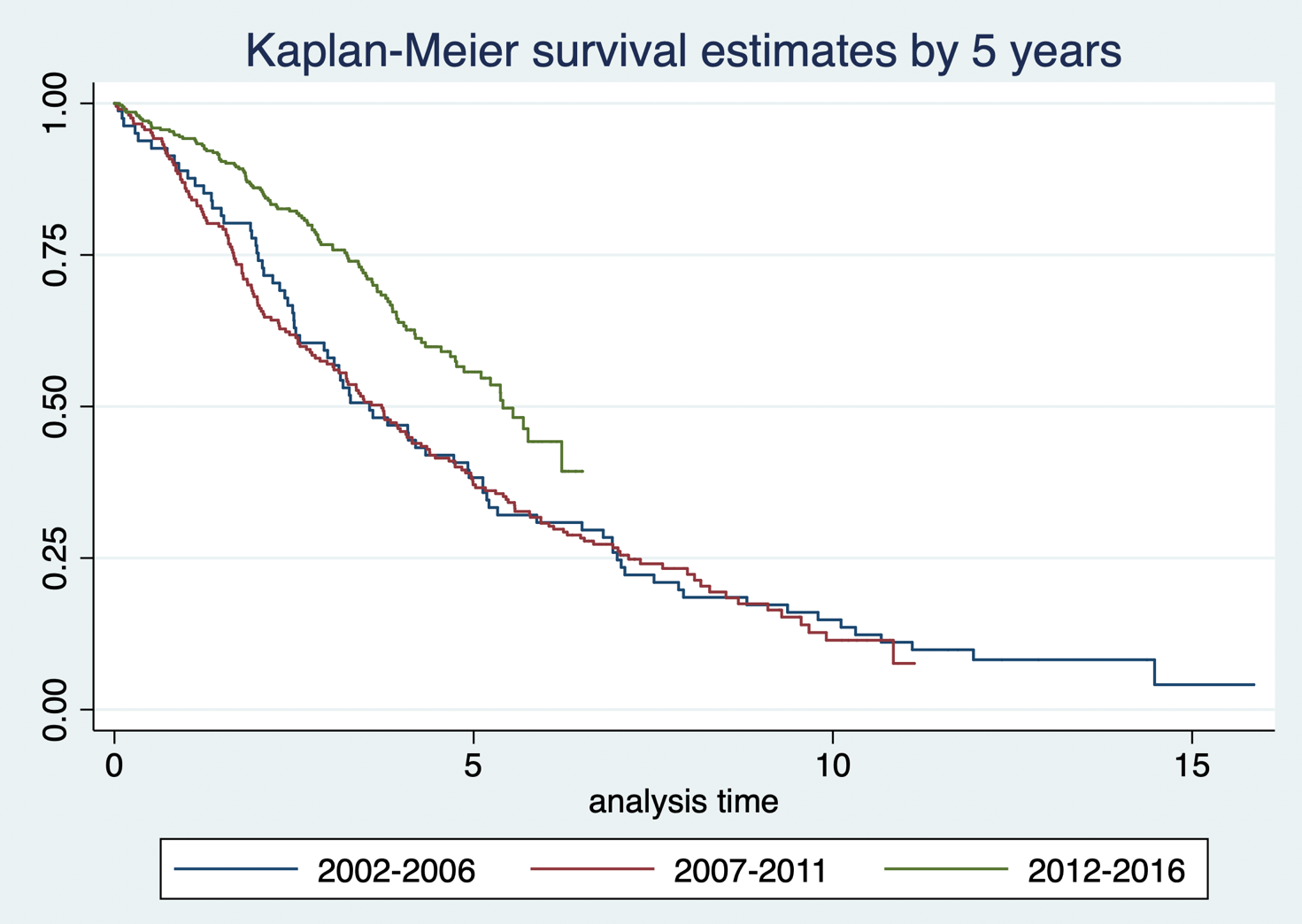


Table S1 propensity score matched analysis for treatment effect on lung cancer risk

| Outcome | Treatment | ATE Coeff | SE | p-value |
| --- | --- | --- | --- | --- |
| Lung Cancer | any antifibrotic | -0.03 | 0.03 | 0.4 |
|  | pirfenidone | -0.03 | 0.03 | 0.2 |
|  | nintedanib | -0.02 | 0.03 | 0.4 |
|  | any immunosoppressive agent | -0.04 | 0.05 | 0.4 |
|  | corticosteroids only | 0.1 | 0.06 | 0.1 |

ATE: average treatment effect; SE: AI Robust Standard Error

Figure S6 PS match graph for treated and untreated


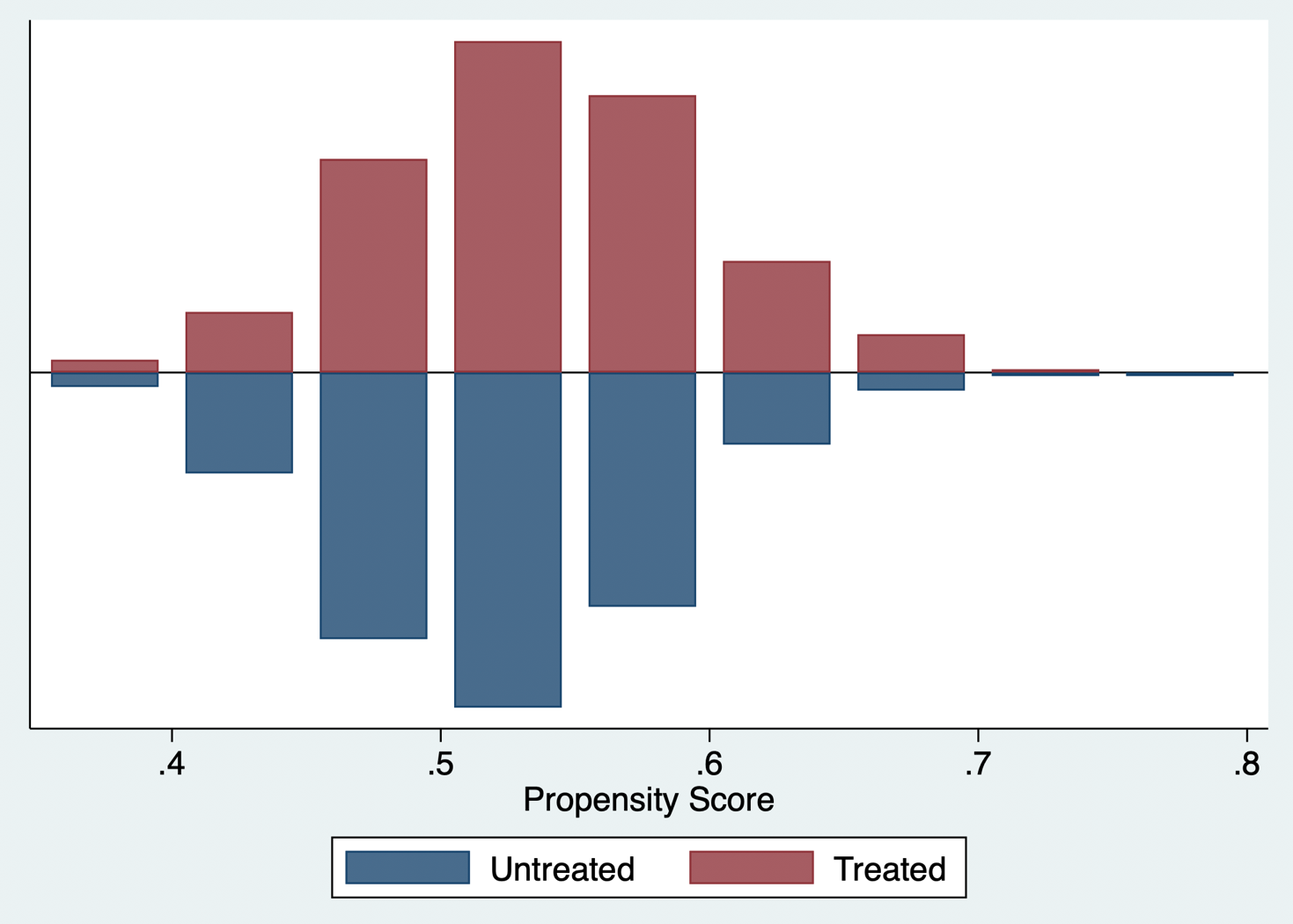

Supplement: Supplementary file 1 [file Data_Sheet_1.docx]
